# The paracrine effects of tumor-resident mesenchymal stem/stromal cells in the microenvironment of brain metastases

**DOI:** 10.3389/fonc.2026.1891707

**Published:** 2026-07-09

**Authors:** Tamer Ayberk Kaya, Klaus-Peter Stein, Belal Neyazi, Ali Rashidi, Ulf Dietrich Kahlert, Christian Mawrin, Ibrahim Erol Sandalcioglu, Claudia Alexandra Dumitru

**Affiliations:** 1 Department of Neurosurgery, Otto-von-Guericke University, Magdeburg, Germany; 2 Molecular and Experimental Surgery, University Clinic for General-, Visceral-, Vascular- and Transplantation Surgery, Otto-von-Guericke University, Magdeburg, Germany; 3 Research Campus STIMULATE, Otto-von-Guericke University, Magdeburg, Germany; 4 Department of Neuropathology, Otto-von-Guericke University, Magdeburg, Germany

**Keywords:** angiogenesis, brain metastases, mesenchymal stem/stromal cells, neutrophil granulocytes, tumor progression

## Abstract

**Background:**

Tumor-resident mesenchymal stem/stromal cells (MSCs) were identified in several cancers, and were implicated in regulating multiple aspects of tumor progression. However, the role of tumor-resident MSCs in the pathophysiology of brain metastases (BrMs) -the most prevalent type of malignant brain tumor- is currently unknown, and was investigated in this study.

**Methods:**

Tumor-resident MSCs were isolated from fresh human lung-BrM tissues (n=3) and were phenotypically assessed in FFPE lung-BrM tissues (n=11). The paracrine effects of BrM-MSCs on neutrophil granulocytes and endothelial cells (ECs) were examined in *ex vivo* models using MSC-derived supernatants (SNs), peripheral blood neutrophils from healthy donors and two different EC models (HUVEC and HCAEC).

**Results:**

The isolated BrM-MSCs were characterized and validated according to the ISCT^®^ criteria. Cells with an MSC-like phenotype were also identified *in situ*, in the majority of the samples analyzed (9 out of 11). The MSC-derived SNs promoted neutrophil chemotaxis, and prolonged their survival. Additionally, BrM-MSC SNs stimulated neutrophils to release significant levels of pro-angiogenic and pro-invasive matrix metalloprotease (MMP) 9. In contrast, BrM-MSC SNs had no direct stimulatory effect on the ECs regarding MMP2 release, migration and proliferation, and even inhibited the tubulogenesis in HUVEC. However, the latter effect could not be validated with the HCAEC cells.

**Conclusion:**

These results indicate that BrMs harbor tumor-resident MSCs that modulate neutrophil biology and function within the BrM microenvironment. While the direct effects of BrM-MSCs on ECs remain to be further characterized, tumor-resident MSCs may play critical roles in the pathophysiology of this disease.

## Introduction

Brain metastases (BrMs) are the most common malignant tumors of the central nervous system, occurring about ten times more frequently than primary brain tumors, such as glioblastoma (1). BrMs originating from lung cancer, in particular non-small-cell lung cancer (NSCLC) is the most prevalent type of BrMs (2). At least half of the NSCLC patients develop BrMs. Within this group, 15-20% of patients already present with BrM at first diagnosis and up to 40% develop them during disease progression (3). BrMs are associated with poor prognosis and high morbidity. Without immediate treatment, the median survival of patients with NSCLC-BrMs is approximately seven months (4). Combined modality therapies have prolonged the survival and improved the quality of life in these patients, yet therapy resistance and recurrence remain as major obstacles. These challenges underscore the need for a better understanding of the BrM pathophysiology, which may ultimately lead to the development of more effective therapeutic strategies for this disease.

Mesenchymal stem/stromal cells (MSCs) are multipotent cells characterized by cell surface expression of CD90, CD105 and CD73, while lacking hematopoietic cell markers, such as CD45 (5). Their “stemness” is defined by their ability to differentiate into other cell types, including adipocytes, chondrocytes and osteoblasts. MSCs generally have a low immunogenicity, due to the negligible expression of major histocompatibility complex (MHC) molecules (6). Therefore, the main body of the MSC research focused on the application of these cells, in particular of bone marrow-derived MSC, as cell-based therapeutics for a variety of medical conditions, including cancer (7). However, accumulating evidence indicates that MSCs also reside directly in the tumor microenvironment (TME) (reviewed in (8, 9)). It has been proposed that naïve MSCs migrate from the surrounding healthy tissues (10, 11) or the systemic circulation (12) to the tumor site, where they can be genetically and functionally reprogrammed by interacting with the TME (13). Subsequently, these cells may facilitate tumor progression by stimulating angiogenesis (14, 15), enhancing tumor growth (16), promoting invasion and metastasis (17), as well as contributing to immunotherapy resistance (18). Importantly, tumor-resident MSCs also exhibit potent immunomodulatory functions. For instance, in spontaneous lymphoma, MSCs were shown to enhance the recruitment of myeloid cells to the TME, which subsequently promoted tumor growth (19). In breast cancer, MSCs induced tumor progression via differentiation of monocytic myeloid-derived suppressor cells (m-MDSCs) into highly immunosuppressive M2 macrophages (20), while in multiple myeloma, they were reported to enhance the immunosuppressive functions of granulocytic MDSCs (21). Furthermore, Gao et al. recently showed that MSCs suppressed the anti-tumor activities of NK and T cells, thereby protecting tumor cells from immune destruction in lung cancer (22).

The role of tumor-resident MSCs in the pathophysiology of BrMs is, however, currently unknown. In this study, we aimed to 1) isolate and characterize MSCs from human NSCLC-BrM tissues, 2) assess their immunomodulatory effects on the biology and function of neutrophil granulocytes and, 3) evaluate their impact on critical steps of the angiogenic cascade, including basal membrane degradation, migration, proliferation, and tubulogenesis of the endothelial cells (ECs).

## Materials and methods

### Materials

All material used in this study, including manufacturer and catalog number, are provided in **Supplementary Table 1**.

### Study subjects

This study included only adult patients with histopathologically confirmed BrMs. Tumor-resident MSCs were isolated from the tissues of three patients with NSCLC-BrM treated at the University Hospital Magdeburg in 2022 and 2023. The study was approved by the ethics committee of the Otto-von-Guericke University Magdeburg (NeuroCAM study 145/19), and informed written consent was obtained from each participant. The *in situ* characterization of tumor-resident MSCs was performed on formalin-fixed paraffin-embedded (FFPE) tumor tissues derived from 11 patients with NSCLC-BrM, who had been treated at the same hospital between 2017 and 2019. This set of studies was also approved by the local ethics committee (NeuroBIOM study 146/19). The committee additionally waived the need for informed consent, since these analyses were performed on leftover material stored at the Department of Neuropathology, University Hospital Magdeburg after the routine diagnostic procedure. None of the patients included in this study had received any systemic therapy (chemo- or immunotherapy) prior to the resection of the BrMs. All studies were conducted in accordance with the Declaration of Helsinki issued in 1975 and revised in 2013.

### Isolation and characterization of tumor-resident MSCs

Fresh BrM tissues were mechanically and enzymatically dissociated using the Tumor Dissociation Kit for 1 h at 37 °C, according to manufacturer´s instructions. The samples were resuspended in cell culture medium (Dulbecco´s modified Eagle´s medium (DMEM) supplemented with 10% FBS Supreme and 1% penicillin – streptomycin), allowed to adhere to plastic overnight, and then further cultured at 37 °C until reaching full confluence. To verify the purity of the isolated BrM-MSCs and characterize cell surface marker expression, the cells were stained with CD45-PE, CD73-APC, CD90-FITC, and CD105-PE-Vio 770 antibodies or corresponding isotype control antibodies, according to manufacturer´s instructions. The samples were subsequently analyzed by flow cytometry using a BD FACSCanto II cytometer. To evaluate the tri-lineage differentiation potential of the isolated cells, BrM-MSCs were stimulated with Mesenchymal Stem Cell Adipogenic, Chondrogenic, and Osteogenic Differentiation media, according to the manufacturer´s instructions. The subsequent detection of lipid vesicles, cartilage and mineralized bone matrix was performed as previously described (23). To phenotypically characterize tumor-resident MSCs *in situ*, the FFPE BrM tissues were cut into 4 µm sections, deparaffinized, and subjected to heat-induced epitope retrieval (HIER) in citrate buffer pH 6.0. Multiplex immunofluorescence was subsequently performed using the Opal-6-Plex kit according to the manufacturer’s instructions and as previously described (23).

### Cell lines and conditioned supernatants

The EC lines used in this study, HUVEC and HCAEC, were kindly provided by the Department of Cardiology and Angiology, Otto-von-Guericke University Magdeburg, but are also commercially available. Conditioned supernatants (SNs) were produced from BrM-MSCs seeded at a density of 5x10^5^ cells/mL in cell culture medium for 24 h at 37 °C. All BrM-MSCs were in passages 3 to 6 and had a confluence of around 90% prior to setting the conditioned SNs. The SNs were finally cleared by centrifugation at 1,200 x g, aliquoted and stored at -20 °C until use. For the neutrophil chemotaxis assay, the BrM-MSC SNs were diluted 1:3 v/v with regular cell culture medium. For all other assays, the SNs were diluted 1:1 v/v.

### Western blot

BrM-MSCs were lysed on ice using Cell Lysis Buffer supplemented with Protease and Phosphatase Inhibitor Cocktail. After removal of the cell debris by high-speed centrifugation, lysates were mixed with SDS sample buffer containing 4% glycerin, 0.8% SDS, 1.6% beta-mercaptoethanol and 0.04% bromophenol blue, and were then subjected to electrophoretic separation by SDS-PAGE. The proteins were subsequently transferred to Roti^®^-Fluoro PVDF membranes, blocked with 5% milk, and incubated with monoclonal primary antibodies against GAPDH (0.1 µg/mL), Vimentin (0.11 µg/mL), and αSMA (0.007 µg/mL) overnight at 4 °C. Secondary reactions were performed using AlexaFluor^®^-488, AlexaFluor^®^-555, and AlexaFluor^®^-647-conjugated antibodies for 1 h at room temperature. All antibodies were diluted with the Signal Boost™ Immunoreaction Enhancer Kit, and signal detection was performed with a ChemoStar imaging system.

### Neutrophil isolation

Neutrophils were isolated from the peripheral blood of healthy donors as previously described (24). The purity of the isolated neutrophils was assessed on a routine basis, and was found to be ≥ 98%.

### Chemotaxis assay

Neutrophil chemotaxis was assessed using transwell inserts with 3 µm pores. The wells of a 24-well plate were filled with 800 µl BrM-MSC SNs or cell culture medium, and inserts were placed into the wells. The neutrophils were then loaded into each insert at a density of 5x10^5^ cells/200 µL medium. After an incubation step of 3 h at 37 °C, the numbers of migrated cells were counted in duplicates with a Neubauer chamber. The studies were performed with neutrophils from four independent blood donors, each with one technical replicate.

### Cell death/survival assay

Neutrophils were incubated with BrM-MSC SNs or cell culture medium at a density of 1x10^6^ cells/mL for 24 h at 37 °C. Cell death was assessed using FITC Annexin V Apoptosis Detection Kit with PI, according to manufacturer´s instructions. The samples were analyzed with a BD FACSCanto II flow cytometer. For quantification, the percentage of surviving cells (Annexin V-negative/PI-negative) was used. The studies were performed with neutrophils from five independent blood donors, each with one technical replicate.

### Gelatine zymography

To determine the levels of gelatinases (MMP2 and MMP9), neutrophils were incubated with BrM-MSC SNs or cell culture medium at a density of 1x10^6^ cells/mL for 1h at 37 °C. The cells were removed by centrifugation, and the resulting supernatants were subsequently mixed with zymogram sample buffer at a final concentration of 80 mM Tris pH 6.8, 1% SDS, 4% glycerol and 0.006% bromophenol blue. The proteins were separated by SDS-PAGE containing 0.2% gelatin 180 Bloom, and were then renaturated in 2.5% Triton-X-100 for 1 h at room temperature. The enzymatic reaction was carried out in a buffer containing 50 mM Tris pH 7.5, 200 mM NaCl_2_ and 1% Triton-X-100 overnight at 37 °C. The gels were then stained with a solution containing 0.5% Brilliant Blue G250, 30% methanol and 10% acetic acid for 1 h at room temperature, followed by three de-staining steps with 10% acetic acid and 30% methanol until the digested bands became visible. The bands were analyzed with the ImageJ 1.48v software. The studies were performed with neutrophils from four independent blood donors, each with one technical replicate.

The same procedure was performed for ECs, except that ECs were first stimulated with BrM-MSC SNs for 3 days, washed, and then allowed to release gelatinases in regular cell culture medium for another 3 days at a density of 1x10^5^ cells/mL. These studies included four independent experiments/biological replicates, each with one technical replicate.

### Cytokine analysis

The levels of CCL4 released by neutrophils, as well as the concentrations of IL-6, IL-8, TNFα, and TGF-β1 in the BrM-MSC SNs, were determined using DuoSet^®^ ELISA kits, according to the manufacturer´s recommendations. The levels of ceruloplasmin (CP) in the BrM-MSC SNs were assessed with a CP ELISA Kit, according to manufacturer´s instructions. The OD values were measured at 450/540 nm on a TECAN plate reader. The studies were performed with neutrophils from three independent blood donors, each with two technical replicates.

### Migration assay

The migration of ECs was measured using the Oris™ system. Initially, 96-well plates were coated with a matrix containing 1 mg/mL collagen I for 30 min at 37 °C, and the cells were seeded in the presence of a stopper to create a cell-free “gap” in the middle of the wells. After adherence overnight, the stoppers were removed, and the cells were allowed to migrate in the cell-free area for 24 h. The degree of “gap closure” (cell-free area at 0h vs 24 h) was quantified using the ImageJ 1.48v software. These studies included four independent experiments/biological replicates, each with two technical replicates.

### Proliferation assay

The proliferation of ECs was measured using the MTT assay. Initially, the cells were seeded in 96-well plates at a density of 4000 cells/well. At the specified time points, cell culture medium containing 10% Thiazol Blue (3-(4,5-demithylthiazol-2-yl)-2,5-diphenyltetrazolium bromide) was added for 4 h at 37 °C to allow formation of formazan crystals. Subsequently, the cells were lysed with isopropanol containing 0.33% HCl, and the OD values were measured at 540/690 nm on a TECAN plate reader. These studies included three independent experiments/biological replicates, each with 8 technical replicates.

### Tube formation assay

The tubulogenesis of ECs was evaluated using tube formation assays. Briefly, 96-well plates were coated with 50 µL undiluted Cultrex Basement Membrane Extract for 30 min at 37 °C. The cells were then seeded at a density of 1.5x10^4^ cells/well, followed by an incubation step of 5 h at 37 °C. The number and length of segments and branches were quantified using the angiogenesis macro of the ImageJ 1.48v software. These studies included four (for HUVEC) and three (for HCAEC) independent experiments/biological replicates, each with two technical replicates.

### Statistical analysis

All data were statistically analyzed using the paired Student´s t-test. To evaluate the paracrine effects, each BrM-MSC supernatant was compared directly to its corresponding negative control (regular cell culture medium) in independent pairwise comparisons using matched donor samples. The level of significance was set at p ≤0.05 in all studies.

## Results

### Isolation and characterization of BrM-MSCs

**Figure 1:**
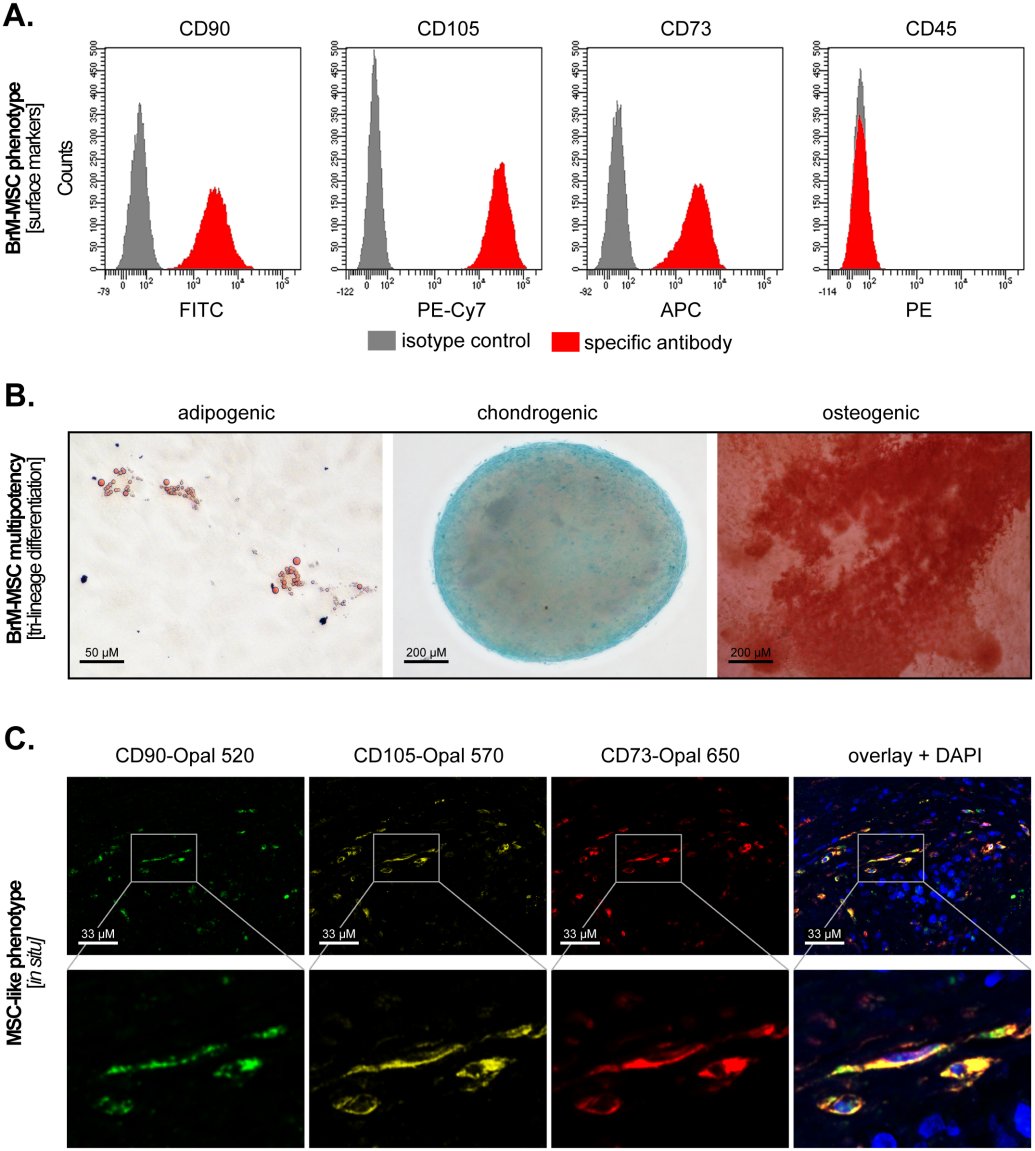
Characterization of BrM-derived MSCs *ex vivo* and *in situ*. **(A)** Representative flow cytometric analysis of showing surface marker expression of CD90, CD105, CD73 and CD45. The specific stainings are indicated by red histograms and the respective isotype controls by grey histograms. All three BrM-MSCs included in our study expressed this phenotypic profile. **(B)** Representative micrographs of the tri-lineage differentiation of BrM-MSCs’ tri-lineage differentiation assay showing lipid vesicles stained with Sudan III (left panel), cartilage stained with Alcian blue (middle panel), and mineralized bone matrix stained with Alizarin red (right panel). All three BrM-MSCs included in our study underwent this tri-lineage differentiation. **(C)** Representative multiplex immunofluorescence micrographs of NSCLC-BrM tissues stained against CD90 (green), CD105 (yellow) and CD73 (red) showing cells positive for all three markers. The nuclei were counterstained with DAPI (blue). These triple-positive cells were identified in 9 out of 11 NSCLC-BrM tissues.

To elucidate the effects of tumor-resident MSCs on the pathophysiology of BrM, we first isolated MSCs from tumor tissues obtained from three different NSCLC-BrM patients. The phenotypic characterization of the isolated cells by flow cytometry showed a consistently high expression of CD90, CD105 and CD73, while the hematopoietic cell marker CD45 was negative (**Figure 1A**). Furthermore, fluorescent western blot analysis confirmed the expression of mesenchymal markers Vimentin and αSMA for all three BrM-MSCs (**Supplementary Figure 1**). To assess multipotency, we performed tri-lineage differentiation assays using lineage-specific stimulation media. The results showed that the cells successfully differentiated into the adipogenic, chondrogenic and osteogenic lineages upon stimulation (**Figure 1B**), though the adipogenic potential was relatively low compared to the other two lineages. We also sought to determine whether cells with an MSC-like phenotype could be actually identified *in situ*. To this end, we stained FFPE tumor tissues from 11 NSCLC-BrM patients by multiplex immunofluorescence against the MSC markers CD90, CD105 and CD73. We found that the majority of samples (9 out of 11) contained triple-positive cells, which were mostly localized in the tumor stroma (**Figure 1C**). Taken together, these findings indicate that the BrM microenvironment contains tumor-resident MSCs, which can be isolated and cultured *ex vivo*.

### Effect of BrM-MSCs on neutrophil biology and function

**Figure 2:**
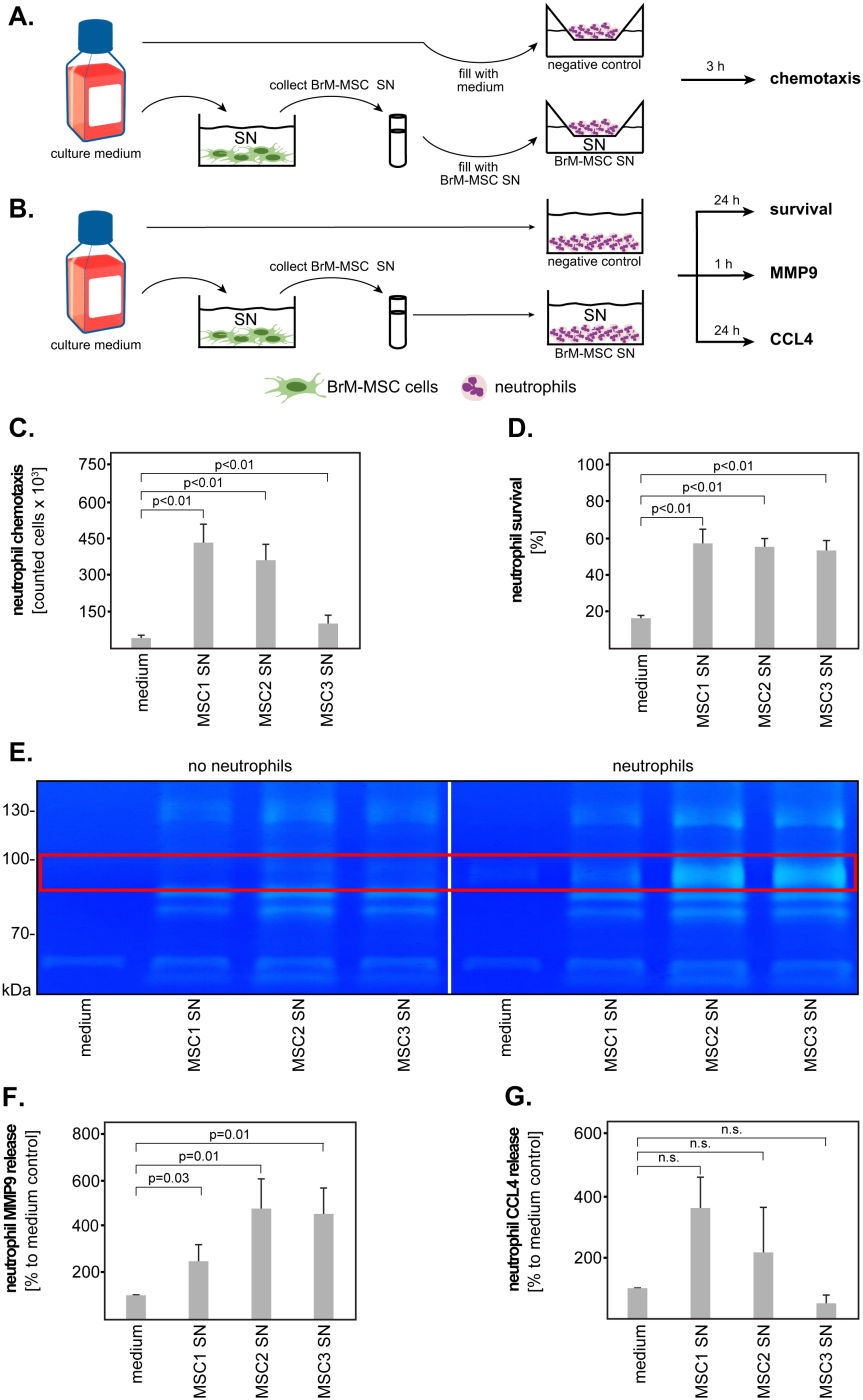
Effect of BrM-MSCs on neutrophil recruitment and pro-tumor phenotype. **(A)** Schematic representation of the neutrophil recruitment (chemotaxis) assay. BrM-MSC SNs were used as chemoattractants, and the migration of neutrophils toward these SNs was assessed in a transwell system. As negative control, neutrophils were allowed to migrate towards regular cell culture medium. **(B)** Neutrophils were stimulated with BrM-MSC SNs for the indicated time points and tested for survival, release of MMP9 and release of CCL4. Neutrophils incubated in regular cell culture medium served as negative control in all three assays. **(C)** All three BrM-MSC SNs had a significant chemotactic effect on neutrophils compared to medium control. **(D)** All three BrM-MSC SNs significantly prolonged the survival of neutrophils. **(E)** Representative gelatin zymography of neutrophils stimulated with BrM-MSC SNs (right panel). The endogenous gelatinase activity of the BrM-MSC SNs in the absence of neutrophils is shown in the left panel. Neutrophil-derived MMP9 is indicated by a red frame. **(F)** Neutrophils stimulated with BrM-MSC SNs released significantly more MMP9 than those from the medium control group. **(G)** The release of CCL4 by neutrophils stimulated with BrM-MSC SNs versus medium control. Neutrophils from three **(G)**, four **(C, F)** and five **(D)** independent donors were included in the respective assays. Shown are the means + S.D. All statistical analyses were performed with the paired t-test.

To investigate the role of BrM-MSCs in neutrophil recruitment (chemotaxis), we prepared conditioned SNs from the isolated BrM-MSCs and used them as chemoattractants in a transwell system (**Figure 2A**). As control, neutrophils were allowed to migrate toward regular cell culture medium (DMEM + 10% FBS). All three BrM-MSC SNs had a significantly increased chemotactic effect on neutrophils compared to the medium control (**Figure 2C**). Next, we stimulated neutrophils with the same BrM-MSC SNs to investigate whether they acquired a tumor-promoting phenotype, characterized by prolonged survival, as well as by the release of pro-invasive/pro-angiogenic (MMP9) and pro-inflammatory (CCL4/MIP-1β) factors (**Figure 2B**). Neutrophils stimulated with regular cell culture medium served as control in all assays. The effect of BrM-MSC SNs on neutrophil survival was tested by Annexin–V/propidium iodide staining, and subsequently quantified by flow cytometry at 24 h post-stimulation. All three BrM-MSC SNs significantly prolonged the survival of neutrophils compared to medium control (**Figure 2D**). Representative flow cytometry plots of the Annexin-V/PI staining in neutrophils stimulated with BrM-MSC SNs versus medium control are shown in **Supplementary Figure 2A**. To assess MMP9 release, neutrophils were stimulated with BrM-MSC SNs or regular cell culture medium for 1 h, and the resulting supernatants were analyzed by gelatin zymography (**Figure 2E** - right panel). Since MSCs can also release MMPs, we additionally analyzed the MMP9 levels in the absence of neutrophils (**Figure 2E** – left panel). The final MMP9 levels were calculated by subtracting the BrM-MSC-derived MMP9 from the neutrophil-derived MMP9. All three BrM-MSC SNs induced neutrophils to release significantly more MMP9 than regular cell culture medium (**Figure 2F**). The full-length gels of all zymography assays are shown in **Supplementary Figure 2B**. To determine whether neutrophils acquired a pro-inflammatory phenotype upon stimulation with BrM-MSC SNs, we measured the levels of neutrophil-derived CCL4 by ELISA at 24h post-stimulation. CCL4 was chosen as a representative factor since it is known to be produced by neutrophils, but it was not present in our BrM-MSC SNs. The results showed that two out of three BrM-MSC SNs promoted the release of CCL4 by neutrophils compared to medium control. However, statistical significance was not reached in any of the samples (**Figure 2G**). To explore the potential mediators of these effects, we analyzed our BrM-MSC SNs by ELISA, with particular focus on cytokines known to modulate the biology and functions of neutrophils. The results showed that BrM-MSCs released very high levels of IL-6 (19.3 ± 19.6 ng/mL) and IL-8 (23 ± 21.6 ng/mL). We also found relatively high levels of CP in these SNs (549 ± 636.5 pg/ml). Other tested cytokines, such as TNFα and TGF-β1 were under the detection limit of the respective assays (**Supplementary Figure 3**). Together, these findings indicate that BrM-MSCs release soluble mediators, which recruit neutrophils to the tumor microenvironment, prolong their survival and induce them to release tumor-promoting factors, in particular MMP9.

### Effect of BrM-MSCs on angiogenesis

**Figure 3:**
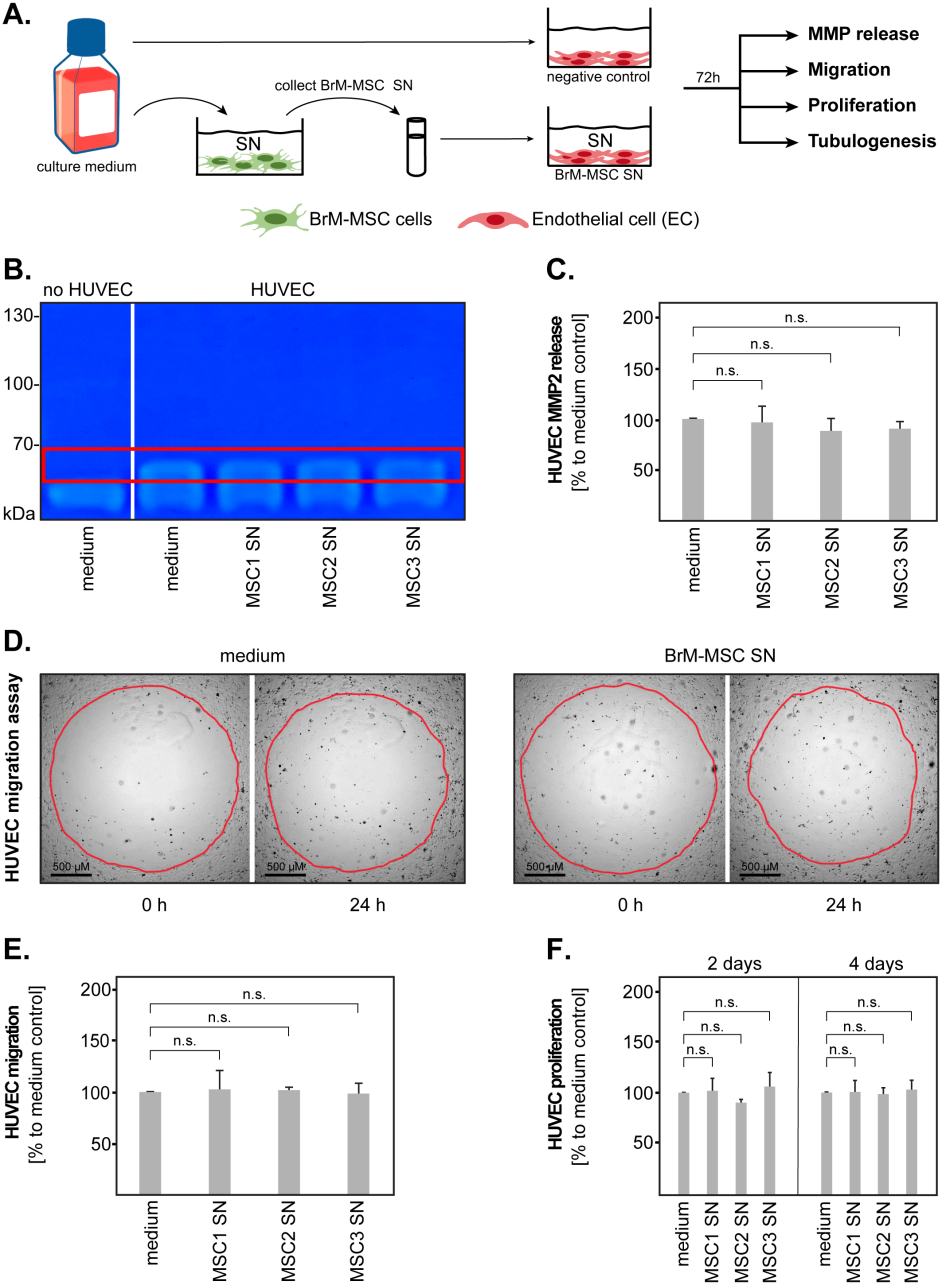
Effect of BrM-MSCs on EC-induced matrix degradation, migration and proliferation. **(A)** Schematic representation of EC stimulation with BrM-MSC SNs and subsequent analysis. ECs incubated in regular cell culture medium served as negative control in all assays. **(B)** Representative gelatin zymography of HUVEC stimulated with BrM-MSC SNs versus medium control. Cell culture medium without HUVEC was used as negative control. HUVEC-derived MMP2 is indicated by a red frame. **(C)** BrM-MSC SNs did not significantly increase the release of MMP2 by HUVEC. **(D)** Representative micrographs of HUVEC migration at 0 h (pre-migration status) and 24 h (post-migration status). The red lines mark the closure of the “gap”, indicating the degree of cell migration. **(E)** BrM-MSC SNs did not significantly promote the migration of HUVEC. **(F)** HUVEC proliferation at 2 days and 4 days post-stimulation, as measured by the MTT assay. BrM-MSC SNs did not significantly enhance the proliferation of HUVEC. Shown are the means + S.D. of three **(F)** and four **(C, E)** independent experiments. All statistical analyses were performed with the paired t-test.

Next, we investigated whether MSCs may affect the angiogenic processes in the BrM microenvironment. To this end, we prepared again conditioned SNs from the BrM-MSCs and stimulated the ECs for 72h. We then examined several key steps in the angiogenic cascade, namely the degradation of the basal membrane (as determined by the release of MMPs by ECs), as well as the EC migration, proliferation, and tubulogenesis (**Figure 3A**). ECs stimulated with regular cell culture medium (DMEM + 10% FBS) served as control in all assays. For this set of studies, we initially used HUVEC as an EC model. The release of MMPs by ECs was assessed at 3 days post-stimulation by gelatin zymography. The results showed that HUVEC released MMP2, but not MMP9 (**Figure 3B**). However, there was no significant difference in MMP2 release between HUVEC stimulated with BrM-MSC SNs and those from the medium control group (**Figure 3C**). EC migration was assessed at 24 h post-stimulation using the Oris™ system (**Figure 3D**). BrM-MSC SNs showed no significant effect on the “gap closure” of HUVEC compared to medium control, as indicated by the ImageJ analysis of the cell-free area at 0h (pre-migration status) vs 24 h (post-migration status) (**Figure 3E**). To assess EC proliferation, we performed MTT assays at 2 and 4 days post-stimulation, but found no significant differences in the metabolic activity of HUVEC stimulated with BrM-MSC SNs compared to medium control (**Figure 3F**). Similar results regarding these three functional read-outs were obtained using the second EC model, HCAEC (data not shown).

**Figure 4:**
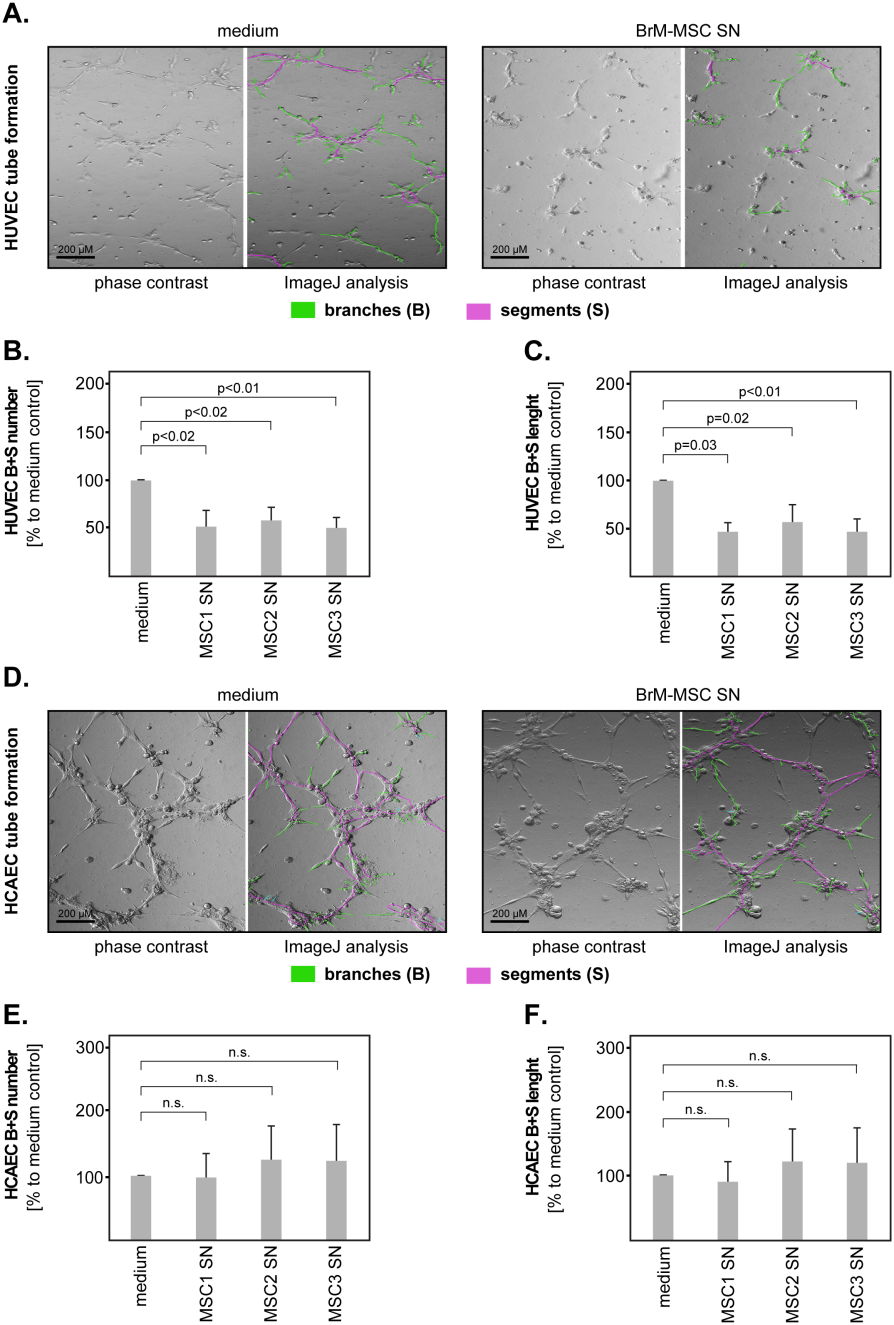
Effect of BrM-MSCs on EC tubulogenesis. **(A)** ImageJ analysis of the tube formation assays in HUVEC stimulated with BrM-MSC SNs (right panels) versus medium control (left panels). The branches are shown in green and the segments in magenta. All three BrM-MSC SNs significantly reduced the **(B)** number and **(C)** length of branches and segments in HUVEC cells compared to medium control. Shown are the means + S.D. of four independent experiments. **(D)** ImageJ analysis of the tube formation assays in HCAEC stimulated with BrM-MSC SNs (right panels) versus medium control (left panels). The branches are shown in green and the segments in magenta. BrM-MSC SNs did not significantly affect the **(E)** number and **(F)** length of branches and segments in HCAEC compared to medium control. Shown are the means + S.D. of three independent experiments. All statistical analyses were performed with the paired t-test.

To assess EC tubulogenesis, we performed tube formation assays, and quantified the development of branches and segments using the ImageJ software at 5 h post-stimulation (**Figure 4A**). All three BrM-MSC SNs significantly reduced both the number (**Figure 4B**) and length (**Figure 4C**) of branches and segments in HUVEC compared to medium control. The tube formation assay and analysis of HCAEC stimulated with BrM-MSC SNs were performed in a similar manner to HUVEC. (**Figure 4D**). However, in contrast to HUVEC, there was no significant difference regarding the number (**Figure 4E**) and length (**Figure 4F**) of branches and segments in HCAEC stimulated with BrM-MSC SNs compared to medium control. Overall, these findings suggest that BrM-MSCs have no direct effect on the basal membrane degradation by ECs, or on the EC migration and proliferation, while the effect on EC tubulogenesis remains to be further characterized.

## Discussion

Tumor-resident MSCs have been previously isolated from several types of cancer, and were tested regarding their ability to modulate key pathophysiological processes in these diseases. However, no studies reported the isolation of tumor-resident MSCs from BrM tissues to date. Interestingly, Tew et al. isolated four different cell lines of stromal origin from breast and lung cancer BrM, and termed them “central nervous system metastasis-associated stromal cells” (cMASCs). These four cMASCs highly resembled each other, and revealed a gene expression signature corresponding to cancer-associated fibroblasts, epithelial to mesenchymal transition, and mesenchymal stem cells. Additionally, cMASCs were shown to express high levels of collagen (25). Nonetheless, these cells were broadly characterized without cell type-specific isolation, purification or phenotype characterization. The MSCs isolated in our study met the consensus criteria defined by the International Society for Cellular Therapy (ISCT^®^) (5). Specifically, they adhered to plastic under standard culture conditions, exhibited a CD90^pos^CD105^pos^CD73^pos^CD45^neg^ phenotype, and demonstrated *ex vivo* tri-lineage differentiation capability into adipogenic, chondrogenic, and osteogenic lineages. Although our isolated cells adhered to these ISCT criteria, the additional expression of α-SMA highlights a known phenotypic overlap with cancer-associated fibroblasts (CAFs). Therefore, the minor presence of CAF- or pericyte-like subpopulations within our primary BrM-MSC cultures cannot be entirely excluded. Importantly, we also identified cells with a CD90^pos^CD105^pos^CD73^pos^ phenotype *in situ*, in the majority of the BrM tissues analyzed. Therefore, to the best of our knowledge, our study is the first to isolate and characterize MSCs from BrM tumor tissues.

Our study further aimed to characterize the role of BrM-MSCs in the TME by focusing on their interactions with neutrophils and ECs. A large body of research showed that neutrophils are recruited to the TME through various mechanisms, and are subsequently stimulated to acquire a tumor-promoting phenotype. However, the effect of tumor-resident MSCs on this phenomenon has emerged only in recent years, with only a handful of studies having addressed these interactions thus far. Specifically, Ren et al. showed that tumor-resident MSCs promoted the recruitment of Cd11b^+^Ly6G^+^ neutrophils to the TME in a murine model of spontaneous lymphoma (19). Another study by Zhu and colleagues found that MSCs isolated from gastric cancer tissue modulated neutrophil chemotaxis, survival, activation and function that led to increased secretion of CXCL8, CCL2, oncostatin M, and CCL4 by neutrophils (26). Additionally, Giallongo et al. showed that MSCs isolated from smoldering multiple myeloma activated neutrophils to acquire tumor-promoting functions, as indicated by the inhibition of T-cell proliferation and increased angiogenesis *in vitro* (27). Expanding on prior evidence, our data revealed that BrM-MSCs had a strong chemotactic effect on neutrophils, thereby likely promoting their recruitment to the BrM microenvironment. We further showed that BrM-MSCs prolonged the survival of neutrophils, and induced them to release MMP9. Although statistical significance was not reached, neutrophils stimulated with BrM-MSCs also released higher levels of CCL4 compared to the control group.

To explore the potential factors mediating the effect of BrM-MSCs on neutrophils, we quantified several cytokines and chemokines in the BrM-MSC supernatants, which have been previously reported to modulate the biology and functions of neutrophils. Specifically, we assessed the levels of neutrophil chemoattractants (IL-8 (28, 29) and TNFα (30)), and those of factors known to promote neutrophil survival (CP (30)) or the pro-inflammatory functions of neutrophils (IL-6 (26), TNFα (30) and TGF-β1 (31)). Consistent with the mechanism proposed by Zhu et al. (26), the IL-6 levels in our BrM-MSC SNs mirrored the trend observed regarding the release of pro-inflammatory cytokines (CCL4) by neutrophils. We additionally found a positive correlation between the levels of IL-8 and neutrophil chemotaxis. However, none of the determined cytokines in our samples directly correlated with the neutrophil survival or MMP9 release. It should be mentioned however, that modulation of PMN biology and functions does not occur via a monofactorial mechanism that is driven by individual cytokines and/or receptor activation, but rather a dynamic system involving multiple mediators (30). Thus, while the elevated levels of IL-8, IL-6, and CP in our BrM-MSC supernatants provide a strong, literature-supported rationale for the observed neutrophil modulation, these specific candidate pathways remain speculative and warrant formal functional validation in future studies.

We finally investigated the role of BrM-MSCs on angiogenesis, since MSCs were shown to both promote and inhibit the angiogenic activity in cancer (reviewed in (9, 32, 33)). We did not observe an impact of the BrM-MSCs on the initial steps of the angiogenic cascade, namely basal membrane degradation (MMP release by ECs) or EC migration and proliferation. These findings are rather surprising, since the BrM-MSC SNs used in this study contained high levels of IL-8, a cytokine known to promote angiogenesis (34–36). A potential explanation for this phenomenon is the concomitant release of anti-angiogenic factors by the MSCs, which might counteract the effects of IL-8. Indeed, previous studies showed that MSCs released microRNAs, ROS, and extracellular vesicles, which inhibited various angiogenic steps, including the ECs’ migration, proliferation, colony formation and tubulogenesis (37–40). In line with the latter studies, we did observe an inhibition of EC tubulogenesis using the HUVEC cell line, but these results could not be confirmed using the second EC model (HCAEC). This discrepancy indicates cell type specific variabilities, which may be due to differences in transcriptomic and proteomic profiles of HUVEC and HCAEC, including genes and proteins involved in the angiogenic cascade (41). Furthermore, HCAEC displayed greater susceptibility to inflammation and atherogenesis compared to HUVEC (42), and differed regarding metabolic activity (43). While these findings do not directly explain the differences in tubulogenesis between the two cell lines observed in our study, they suggest that distinct molecular mechanisms may be involved in this phenomenon. Therefore, further studies using additional EC lines or, ideally, primary ECs are necessary to clarify the exact role of tumor-resident MSCs in BrM angiogenesis. In addition to these endothelial and myeloid interactions, tumor-resident MSCs are documented in several malignancies to directly modulate cancer cell invasion, epithelial-to-mesenchymal transition, and therapy resistance via paracrine signaling (reviewed in (33)). Investigating whether BrM-MSCs engage in similar reciprocal cross-talk with metastatic tumor cells will be an essential avenue for future research to fully map the multi-lineage cellular networks driving metastatic progression in the brain.

In summary, our study shows for the first time that BrMs harbor tumor-resident MSCs. These cells could modulate the biology and functions of neutrophils *ex vivo*, in a paracrine manner. Specifically, the MSC-derived factors stimulated neutrophil recruitment/chemotaxis, and induced neutrophils to acquire features resembling a pro-tumor/TAN-like phenotype, as indicated by prolonged survival, and enhanced release of MMP9. However, since our primary BrM-MSCs were isolated from a limited number of patients, these findings should be interpreted with caution regarding the broader clinical heterogeneity of brain metastases. Furthermore, future clinical validation of tumor-resident MSCs utilizing standardized multiplex quantification workflows and automated digital pathology will be essential to fully define the prognostic impact of these cells in BrMs and other types of cancer. While the direct effect of MSCs on BrM angiogenesis still requires characterization, MSCs may nevertheless represent key components of the BrM microenvironment, with critical roles in the pathophysiology of this disease.

## Data Availability

The original contributions presented in the study are included in the article/**Supplementary Material**. Further inquiries can be directed to the corresponding authors.
