# Supplementary material for: The paracrine effects of tumor-resident mesenchymal stem/stromal cells in the microenvironment of brain metastases
Source: Front Oncol. 2026 Jul 9;16:1891707. doi: 10.3389/fonc.2026.1891707 (PMC13391273; doi:10.3389/fonc.2026.1891707)
Supplement: Supplementary file 1 [file DataSheet1.pdf]

Supplementary Table S1. List of materials used in the study according to category.

		Manufacturer	Product No.
Antibodies	AlexaFluor®488 anti-rat IgG F(ab)2	Cell Signaling Technology, Frankfurt am Main, Germany	4416
	AlexaFluor®555 anti-mouse IgG F(ab)2	Cell Signaling Technology, Frankfurt am Main, Germany	4409
	Alexa Fluor® 647 anti-human CD66b	Biolegend, San Diego, CA, USA	305109
	Alexa Fluor® 647 anti-mouse IgG1	Biolegend, San Diego, CA, USA	400155
	AlexaFluor®647 anti-rabbit IgG F(ab)2	Cell Signaling Technology, Frankfurt am Main, Germany	4414
	CD45-PE, anti-human REAfinity	Miltenyi Biotech, Bergisch Gladbach, Germany	130-110-632
	CD73-APC (REA804) anti-human REAfinity	Miltenyi Biotech, Bergisch Gladbach, Germany	130-112-061
	CD73 (D7F9A) rabbit monoclonal	Cell Signaling Technology, Frankfurt am Main, Germany	13160
	CD90-FITC anti-human REAfinity	Miltenyi Biotech, Bergisch Gladbach, Germany	130-114-901
	CD90 (Thy1) rabbit monoclonal	Cell Signaling Technology, Frankfurt am Main, Germany	13801
	CD105/Endoglin (3A9) mouse monoclonal	Cell Signaling Technology, Frankfurt am Main, Germany	14606
	CD105-PE-Vio770 anti-human REAfinity	Miltenyi Biotech, Bergisch Gladbach, Germany	130-112-325
	GAPDH Ab (W17079A) rat monoclonal	Biolegend, San Diego, CA, USA	607901
	REA control antibody, human, APC	Miltenyi Biotech, Bergisch Gladbach, Germany	130-113-446
	REA control antibody, human, FITC	Miltenyi Biotech, Bergisch Gladbach, Germany	130-113-449
	REA control antibody, human, PE	Miltenyi Biotech, Bergisch Gladbach, Germany	130-113-450
	REA control antibody, human, PE-Vio770	Miltenyi Biotech, Bergisch Gladbach, Germany	130-113-452
	Vimentin mouse monoclonal	Proteintech, Rosemont, IL, USA	60330-1-lg
	α-SMA (Smooth Muscle Action) (D4K9N) rabbit monoclonal	Cell Signaling Technology, Frankfurt am Main, Germany	19245
Kits	FITC Annexin V apoptosis detection kit with PI	BioLegend, San Diego, CA, USA	640914
	Human CCL4/MIP-1 beta DuoSet ELISA	RnD Systems, Minneapolis, MN, USA	DY271-05
	Human Ceruloplasmin (CP) ELISA Kit	Cloud-Clone Corp., Katy, TX, USA	SEA909Hu-96T
	Human IL-6/CXCL6 DuoSet ELISA	RnD Systems, Minneapolis, MN, USA	Dy206-05
	Human IL-8/CXCL8 DuoSet ELISA	RnD Systems, Minneapolis, MN, USA	DY208-05

	Human TGF-β1 DuoSet ELISA	RnD Systems, Minneapolis, MN, USA	DY240-05
	Human TNF-α DuoSet ELISA	RnD Systems, Minneapolis, MN, USA	DY210-05
	Opal™ 6-Plex Detection Kit	Akoya Biosciences, Marlborough, MA, USA	NEL811001KT
	Oris™ 3D Embedded Invasion Assay Kit	Platypus Technologies, Madison, WI, USA	EIA1
	Signal Boost™ Immunoreaction Enhancer Kit	Merck-Millipore, Burlington, MA, USA	407207-1KIT
	Tumor Dissociation Kit, human	Miltenyi Biotech, Bergisch Gladbach, Germany	130-095-929
**Chemicals and disposable material**	Acetic acid	Carl Roth, Karlsruhe, Germany	3738.2
	Alcian blue 0,1%, pH 2,5 with acetic acid	Morphisto, Frankfurt, Germany	11490.00250
	Alizarin-red S, pH 4.0	Morphisto, Frankfurt, Germany	13158.00250
	Ammonium peroxydisulphate	Carl Roth, Karlsruhe, Germany	9592.3
	Aqua B. Braun	B. Braun SE, Melsungen, Germany	0082479E
	Beta-mercaptoethanol	Carl Roth, Karlsruhe, Germany	4227.3
	Brilliant blue G 250	Carl Roth, Karlsruhe, Germany	9598.1
	Bromophenol blue	Carl Roth, Karlsruhe, Germany	A512.2
	Cell lysis buffer (10x)	Cell Signaling Technology, Frankfurt am Main, Germany	9803
	Citrate buffer pH 6	Sigma Aldrich, St. Louis, MO, USA	C9999-1000ML
	Cultrex Reduced Growth Factor Basement Membrane Extract	Bio-Techne, Minneapolis, MN, USA	3433-005-01
	Ethanol	Carl Roth, Karlsruhe, Germany	K928.3
	FBS Supreme	Pan Biotech, Aidenbach, Germany	P30-3031
	Gelatine 180 bloom	Carl Roth, Karlsruhe, Germany	4274.4
	Glycerin	Carl Roth, Karlsruhe, Germany	3783.1
	Hydrochloric acid fuming 37%	Carl Roth, Karlsruhe, Germany	X942.1
	Isopropanol (2-propanol)	Morphisto, Frankfurt, Germany	19288.00250
	Mesenchymal Stem Cell Adipogenic Differentiation Medium 2	PromoCell, Heidelberg, Germany	C-28016
	Mesenchymal Stem Cell Chondrogenic Differentiation Medium 2	PromoCell, Heidelberg, Germany	C-28012
	Mesenchymal Stem Cell Osteogenic Differentiation Medium 2	PromoCell, Heidelberg, Germany	C-28013
	Methanol	Carl Roth, Karlsruhe, Germany	0082.2
	Milk powder	Carl Roth, Karlsruhe, Germany	T145.2

	Oris Collagen I (rat Tail)	Platypus Technologies, Madison, WI, USA	24B2801
	Pageruler™ Plus prestained Protein Ladder	Thermo Fisher Scientific, Waltham, MA, USA	26619
	Pancoll human	Pan Biotech, Aidenbach, Germany	P04-60500
	Penicillin-Streptomycin	Thermo Fisher Scientific, Waltham, MA, USA	15140122
	Poly(vinyl alcohol)	Merck, Darmstadt, Germany	P1763-250G
	Protease/phosphatase inhibitor cocktail (100x)	Cell Signaling Technology, Frankfurt am Main, Germany	5872
	ROTI®Cell 10x PBS	Carl Roth, Karlsruhe, Germany	9150.1
	Roti®Cell DMEM high glucose	Carl Roth, Karlsruhe, Germany	9007.1
	Roti®Fluoro PVDF Transfer Membrane	Carl Roth, Karlsruhe, Germany	2803.1
	Rotiphorese®Gel 40 (29:1)	Carl Roth, Karlsruhe, Germany	A515.1
	Saccomanno fixation solution	Morphisto, Frankfurt, Germany	1388100250
	SDS ultra pure	Carl Roth, Karlsruhe, Germany	2326.4
	Sodium chloride	Carl Roth, Karlsruhe, Germany	HN00.2
	Sudan III, alcoholic	Morphisto, Frankfurt, Germany	10396.00250
	TBS (10x)	Thermo Fisher Scientific, Waltham, MA, USA	10776834
	TEMED	Carl Roth, Karlsruhe, Germany	2367.3
	Thiazol blue	Carl Roth, Karlsruhe, Germany	4022.1
	Transwell inserts	Sarstedt, Nümbrecht, Germany	83.3932.300
	Tris	Carl Roth, Karlsruhe, Germany	4855.2
	Triton X-100	Carl Roth, Karlsruhe, Germany	3051.4
	Tween 20®	Carl Roth, Karlsruhe, Germany	9127.1
**Software**	BD FACSDiva™	BD Biosciences, Heidelberg, Germany	n.a.
	ImageJ 1.48v	NIH, Bethesda, MD, USA	n.a.
	Magellan™ 7.2	Tecan Life Sciences, Männedorf, Switzerland	n.a.
**Equipment**	BD FACSCanto II flow cytometer	BD Biosciences, Heidelberg, Germany	n.a.
	BZ-X810 fluorescence microscope	Keyence, Neu-Isenburg, Germany	n.a.
	ChemoStar Imaging system	Intas Science Imaging, Göttingen, Germany	n.a.
	TECAN plate reader	Tecan Life Sciences, Männedorf, Switzerland	n.a.

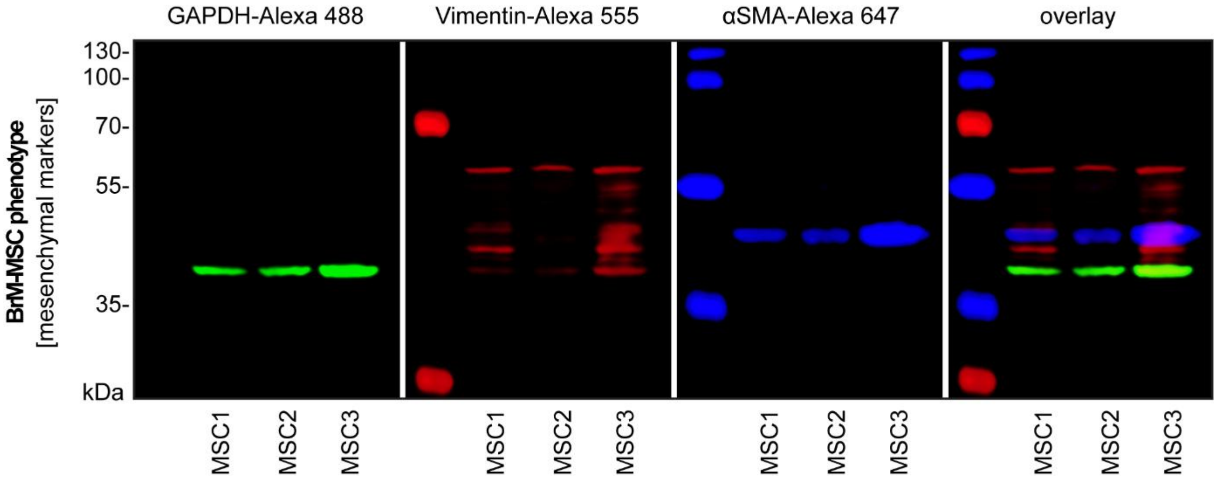

Supplementary Figure S1. Characterization of BrM-derived MSCs *ex vivo*. Fluorescent western blot analysis of all three BrM-MSCs showing expression of the mesenchymal markers Vimentin (red) and αSMA (blue). GAPDH (green) was used as a loading control.

A.

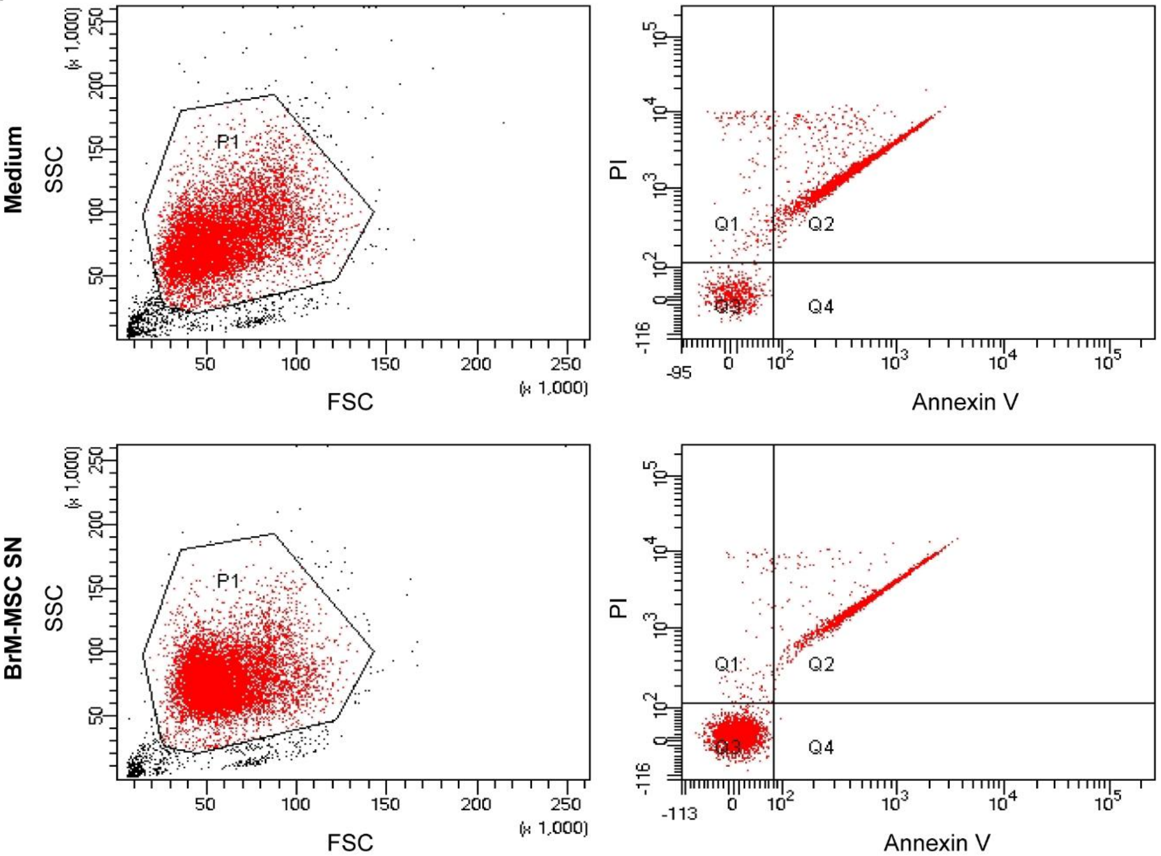

B.

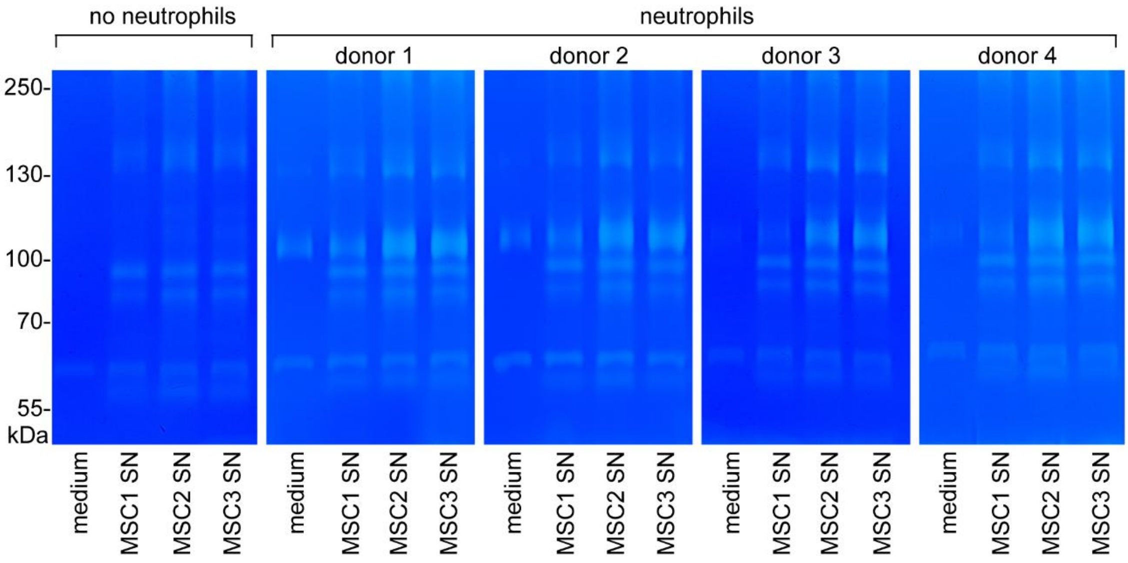

Supplementary Figure S2. Effect of BrM-MSCs on neutrophil pro-tumor phenotype. (A) Representative flow cytometry plots depicting the survival analysis of neutrophils via Annexin-V/PI staining. The surviving cells (Annexin-negative/PI-negative) are indicated in Q3. **(B)** Full-length zymography gels of neutrophils from 4 independent donors stimulated with BrM-MSC SNs (right panels). The endogenous gelatinase activity of the BrM-MSC SNs in the absence of neutrophils is shown in the left panel.

ELISA SAMPLE	IL-6 [ng/ml]	IL-8 [ng/ml]	CP [pg/ml]	TNFα [pg/ml]	TGF-ß1 [pg/ml]
MSC1 SN	39.99	44.79	1280	<15	<31
MSC2 SN	17.02	22.08	250	<15	<31
MSC3 SN	0.99	2.12	117	<15	<31

Supplementary Figure S3. Cytokine levels in BrM-MSC SNs. (G) The levels of IL-6, IL-8, CP, TNFα, and TGF-β1 released by BrM-MSCs, as determined by ELISA.
